# Validation of brief symptom indexes among patients with recurrent or metastatic squamous cell carcinoma of the head and neck: A trial of the ECOG‐ACRIN Cancer Research Group (E1302)

**DOI:** 10.1002/cam4.3506

**Published:** 2020-10-10

**Authors:** Laura B. Oswald, Ju‐Whei Lee, Athanassios Argiris, Kimberly A. Webster, Arlene A. Forastiere, David Cella

**Affiliations:** ^1^ Department of Medical Social Sciences Northwestern University Feinberg School of Medicine Chicago IL USA; ^2^ Department of Data Sciences Dana‐Farber Cancer Institute ECOG‐ACRIN Biostatistics Center Boston MA USA; ^3^ Department of Medical Oncology Thomas Jefferson University Hospital Philadelphia PA USA; ^4^ Department of Oncology Johns Hopkins University Baltimore MD USA

**Keywords:** head and neck cancer, psychosocial studies, quality of life

## Abstract

**Background:**

Patients with advanced head and neck cancer have identified pain, fatigue, and difficulties swallowing, breathing, and communicating as high‐priority disease‐related symptoms. The Functional Assessment of Cancer Therapy‐Head and Neck Symptom Index‐10 (FHNSI‐10) assesses these symptoms. We sought to validate the FHNSI‐10, another brief symptom index (FHNSI‐7), and individual symptom endpoints representing these high‐rated priority disease symptoms among patients with recurrent or metastatic squamous cell carcinoma of the head and neck (SCCHN).

**Methods:**

Patients (N = 239) were enrolled in a phase III randomized clinical trial (E1302) and completed the FHNSI‐10 at multiple time points. We assessed the internal consistencies and test–retest reliabilities of the FHNSI‐10 and FHNSI‐7 scores, and the known‐groups validity, predictive criterion validity, and responsiveness‐to‐change of the symptom indexes and individual symptom endpoint scores.

**Results:**

The FHNSI‐10 and FHNSI‐7 indexes showed satisfactory internal consistencies (Cronbach's alpha coefficient range 0.60‐0.75) and acceptable test–retest reliabilities (intraclass correlation coefficients = 0.75 and 0.74, respectively). The FHNSI‐10, FHNSI‐7, and the pain, fatigue, swallowing, and breathing symptom scores showed evidence of known‐groups validity by performance status at baseline. The FHNSI‐10, FHNSI‐7, and the pain, fatigue, and breathing symptom scores at baseline showed evidence of predictive criterion validity for overall survival, but not time‐to‐progression (TTP). Changes in the symptom indexes and individual symptom scores were not associated with changes in performance status over 4 weeks, though most patients had stable performance status.

**Conclusions:**

There is initial evidence of validity for the FHNSI‐10 and FHNSI‐7 indexes and selected individual symptom endpoints as brief disease‐related symptom assessments for patients with recurrent or metastatic SCCHN.

## Graphical abstract

The patient‐centered FHNSI‐10 and FHNSI‐7 symptom indexes show evidence of validity and reliability for use among patients with recurrent or metastatic squamous cell carcinoma of the head and neck. Individual symptom endpoints (1‐2 items each) are also valid for assessing pain, fatigue, swallowing, and breathing in this population.

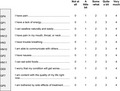

## 1 INTRODUCTION

Head and neck cancer (e.g., cancers of the oral cavity, pharynx, and larynx) accounts for approximately 4% of cancer diagnoses in the United States annually, which translates to more than 53,000 expected new cases in 2019.^1^ Advances in head and neck cancer treatment have resulted in improved survival rates over the past several decades, with the 5‐year relative survival rate for localized head and neck cancer currently estimated as 84%. However, the vast majority (>70%) of patients with head and neck cancer are diagnosed with regional or distant advanced disease, where the 5‐year relative survival rates drop to 65% and 39%, respectively.^1^

Due to their location, these tumors can interfere with vital functions including swallowing, breathing, and speaking. Further, while treatments for advanced head and neck cancer (surgery; radiotherapy; and chemotherapy) may prolong life, they are associated with toxicities that can contribute to even greater symptom burden.^2^, ^3^, ^4^ Assessment of these disease‐ and treatment‐related symptoms is critical for clinical trials in which therapeutic efficacy is evaluated not only by clinical outcomes (e.g., survival and tumor response), but also by patient‐reported outcomes (PROs), such as symptoms and quality of life.^5^

There are several validated instruments available to assess PROs among patients with head and neck cancer,^6^ such as the MD Anderson Symptom Inventory for Head and Neck Cancer (MDASI‐HN)^7^ and the European Organization for Research and Treatment of Cancer Quality of Life Questionnaire Head and Neck module (EORTC‐QLQ‐H&N35).^8^, ^9^ The National Comprehensive Cancer Network‐Functional Assessment of Cancer Therapy (FACT)‐Head and Neck Symptom Index‐22 (NFHNSI‐22) was developed from clinician and patient rankings of priority head and neck cancer concerns and includes items related to symptoms, treatment side effects, and function/well‐being.^10^ With a growing interest in isolating the assessment of specific symptoms, further item reduction to include only the highest priority disease symptoms experienced by patients with advanced head and neck cancer could help promote patient‐centered outcome assessment that is fit for regulatory use. For example, the FACT‐Head and Neck Symptom Index‐10 (FHNSI‐10) includes 10 items from the NFHNSI‐22 that assess high‐priority patient‐reported head and neck cancer symptoms (e.g., pain, fatigue, swallowing, breathing, and communication).^11^, ^12^, ^13^ Even further item reduction will be beneficial.

To address this need, this study sought to validate the scores of very brief symptom indexes for use among patients with advanced head and neck cancer based on prior identification of high‐priority patient‐reported disease symptoms,^11^, ^12^ first as clusters of symptoms (i.e., FHNSI‐10 and FHNSI‐7 symptom indexes) and then as individual symptom endpoints (i.e., pain, fatigue, and difficulty swallowing, breathing, and communicating) among patients with metastatic squamous cell carcinoma of the head and neck (SCCHN) enrolled in a large phase III randomized placebo‐controlled trial (E1302). We evaluated the internal consistencies and test–retest reliabilities of the index scores, and the known‐groups validity, predictive criterion validity, and responsiveness‐to‐change of the symptom index scores and the individual symptom endpoint scores using familiar clinical anchors (i.e., provider‐rated Eastern Cooperative Oncology Group (ECOG) performance status (PS), overall survival, and disease progression). Secondary objectives were to explore the relationships between one additional item assessing overall treatment side effect bother with the symptom index scores, the individual symptom endpoint scores, and provider‐rated adverse events. This work is informed by the conceptualization of the FACT symptom indexes as being causal indicators of symptom burden (vs. effect indicators).^14^, ^15^

## 2 METHODS

### 2.1 Participants and procedures

Participants in this study were enrolled in ECOG‐ACRIN Cancer Research Group Study number E1302, a phase III randomized, placebo‐controlled, double‐blind trial of docetaxel with or without gefitinib to treat recurrent or metastatic SCCHN (ClinicalTrials.gov identifier: NCT00088907).^16^ Eligible patients were at least 18 years old, had been diagnosed with incurable recurrent or metastatic SCCHN, and had a provider‐rated ECOG PS of 0‐2. Exclusion criteria included pregnancy or breastfeeding, recent major tumor‐related hemorrhagic events, current therapeutic anticoagulation, and tumors that had invaded major blood vessels. After providing informed consent, participants were randomized to treatment with docetaxel plus placebo or docetaxel plus gefitinib, and monitored for therapeutic response and disease progression. The primary results of this trial are reported elsewhere.^16^ All protocol procedures were approved by the relevant institutional review boards. Prior to protocol treatment, participants completed a baseline assessment including items assessing head and neck cancer symptoms. Symptom assessments were repeated mid‐way through treatment cycle 1 (Week 2), at the end of treatment cycle 1 (Week 4), and at the end of treatment cycle 2 (Week 8). The data to support the findings of this study are available from the corresponding author upon reasonable request.

### 2.2 Measures

#### 2.2.1 Head and neck cancer symptom assessment

**Figure 1:**
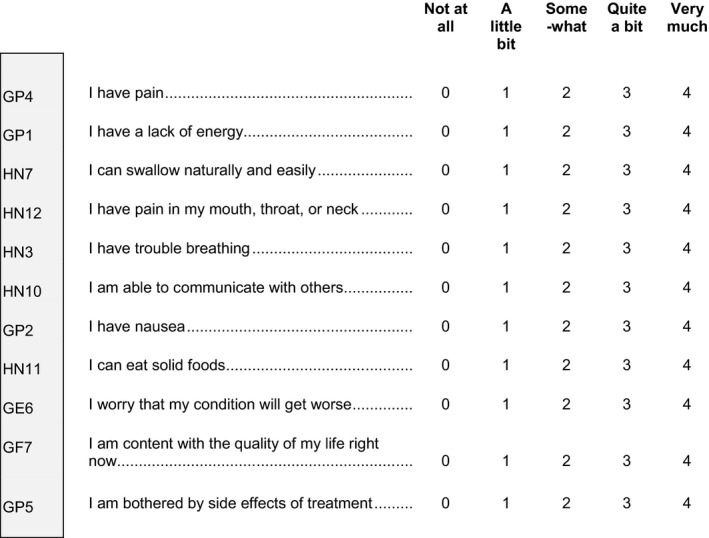
The 10‐item FHNSI‐10 plus one additional item assessing overall treatment side effect bother (GP5) that is not scored with the other items. The following items comprise the FHNSI‐7: GP4, GP1, HN7, HN12, HN3, HN10, and HN11. ©Copyright FACIT.org and reprinted with permission.

Participants completed the 10‐item FHNSI‐10 index of high‐priority patient‐reported head and neck cancer disease symptoms (e.g., pain, fatigue, and difficulties swallowing, breathing, and communicating)^12^, ^13^ and one additional item assessing overall treatment side effect bother (i.e., “I am bothered by side effects of treatment,” item GP5) that is positively associated with clinician‐reported adverse events and negatively associated with patient‐reported enjoyment of life (Figure 1).^17^ Participants rated each item using a 7‐day recall period, on an ordinal rating scale from 0 (*not at all*) to 4 (*very much*). As with all measures in the Functional Assessment of Chronic Illness Therapy (FACIT) system, high scores are better than low scores. Therefore, symptom responses were reversed as necessary, so that high scores represented less pain and fatigue as well as less difficulty swallowing, breathing, and communicating. Consistent with Pearman et al.,^17^ the single treatment side effect bother item (GP5) was not reverse scored and higher GP5 scores indicated more bother from side effects.

From the FHNSI‐10 items, a 7‐item index (“FHNSI‐7”) was computed to include only those items that correspond to symptoms identified in prior research as high‐priority disease‐related symptoms: pain, fatigue, swallowing, breathing, and communication (items GP4, HN12, GP1, HN7, HN11, HN3, and HN10).^11^, ^12^ In addition to the FHNSI‐10 and FHNSI‐7, individual symptom endpoints were computed for each of the following symptoms: pain (items GP4 and HN12); fatigue (item GP1); swallowing (items HN7 and HN11); breathing (item HN3); and communication (item HN10). FHNSI‐10 and FHNSI‐7 scores were computed as the prorated sum of the item responses, provided more than 50% of the items were answered (prorated score=(raw sum*number of total items)/number of items answered). Scores for each individual symptom endpoint were summed, and scores were only computed for patients who had answered all target items for a given symptom.

#### 2.2.2 Anchor variables

Anchor‐based methods were used to evaluate the PRO measures’ known‐groups validity by ECOG PS, predictive criterion validity for meaningful clinical endpoints (i.e., change in ECOG PS, overall survival, and time‐to‐progression (TTP)), and responsiveness‐to‐change by ECOG PS. Patients were classified using the single‐item provider‐rated ECOG PS ranging from 0 (*normal activity without symptoms*) to 4 (*unable to get out of bed*),^18^, ^19^ and change in ECOG PS was defined as ECOG PS at Week 4 minus the value at baseline. Overall survival (OS) was defined as the time from study registration to death from any cause, censored at the date of last contact. TTP was defined as the time from study registration to evidence of disease progression, censored at the date of last disease evaluation.

### 2.3 Statistical analyses

Descriptive statistics were used to characterize the patients and describe the distribution of PRO scores (i.e., FHNSI‐10, FHNSI‐7, and individual symptom endpoints) at baseline and over time. There was no treatment effect on OS in the larger trial,^16^ and there was no main effect of treatment (*p* = 0.11) or a two‐way interaction effect between treatment and time points on FHNSI‐10 scores (*p* = 0.15). Therefore, all PRO scores were combined across treatment groups. All *p*‐values were two‐sided, and a value of <0.05 was considered statistically significant. These analyses were exploratory in nature, so no statistical adjustments were made for tests of multiple comparisons unless otherwise specified.

We calculated Cronbach's alpha coefficients to assess the internal consistency reliability of the FHNSI‐10 and FHNSI‐7 index scores across time, and we calculated intraclass correlation coefficients (ICCs) to assess the test–retest reliability of the FHNSI‐10 and FHNSI‐7 scores from baseline to Week 4 among patients with stable ECOG PS.^20^ We also assessed the known‐groups validity, predictive criterion validity, and responsiveness‐to‐change for the FHNSI‐10 and FHNSI‐7 index scores and the symptom endpoint scores for pain, fatigue, swallowing, and breathing using anchor‐based methods. Of note, we did not assess the validity or reliability of the communication symptom endpoint, as we do not hypothesize that difficulty communicating is related to ECOG PS, OS, or TTP. For known‐groups validity, we used ANOVA tests to differentiate among ECOG PS at baseline with respect to PRO scores, with Scheffe tests to assess post‐hoc pairwise differences. Non‐parametric Kruskal‐Wallis tests were further performed to confirm the ANOVA results for individual symptom endpoint scores. For predictive criterion validity, we evaluated the relationships between baseline PRO scores and longitudinal anchor variables using univariate general linear models (for change in ECOG PS) and Cox proportional hazards (PH) models (for OS and TTP). We used multivariable models to confirm the results of the univariate models adjusting for age, sex, race, disease status, and prior treatments (i.e., chemotherapy, radiotherapy, and surgery, separately),^21^ and we also adjusted for ECOG PS for Cox PH models assessing OS and TTP. We explored the PRO scores’ change over time using mixed linear models with unstructured covariance, with the assessment time point considered as a categorical variable. For responsiveness‐to‐change, we used ANOVA to evaluate the relationships between changes in the PRO scores and changes in ECOG PS, with change scores defined as the value assessed at Week 4 minus the value assessed at baseline. Finally, we used univariate and multivariable general linear models to explore the relationships between the GP5 “bother” item, the PRO scores, and the incidence and severity of provider‐rated adverse events over time.

## 3 RESULTS

### 3.1 Sample characteristics

In total, 270 patients with recurrent or metastatic SCCHN were enrolled in the phase III E1302 trial, and 239 of those patients completed baseline PRO assessments and were eligible for this secondary analysis. See Table 1 for patients’ baseline demographic and disease characteristics. Patients were mostly male (79.5%) and white (84.9%). Notably, most patients had poor prognosis, with a provider‐rated ECOG PS of 2 (62.8%) and prior treatments with chemotherapy (74.5%), radiotherapy (84.9%), and/or surgery (61.1%). Primary head and neck cancer sites were mostly oropharynx (32.6%), larynx (25.5%), or oral cavity (22.2%), and almost half of the patients at baseline had eradicated disease but with local recurrence (46.1%).

**Table 1:** Patient demographic and disease characteristics at study baseline

Patient characteristic	Total sample (N = 239)	Treatment	
		Docetaxel +Gefitinib (N = 122)	Docetaxel +Placebo (N = 117)
Age; median (range)	61.0 (28.0‐86.6)	60.9 (41.6‐84.5)	61.4 (28.0‐86.6)
Sex; n (%)			
Male	190 (79.5)	98 (80.3)	92 (78.6)
Female	49 (20.5)	24 (19.7)	25 (21.4)
Race; n (%)			
White	203 (84.9)	102 (83.6)	101 (86.3)
Non‐white	36 (15.1)	20 (16.4)	16 (13.7)
ECOG PS; n (%)			
0	27 (11.3)	12 (9.8)	15 (12.8)
1	62 (25.9)	31 (25.4)	31 (26.5)
2	150 (62.8)	79 (64.8)	71 (60.7)
Primary head and neck cancer site; n (%)			
Oropharynx	78 (32.6)	42 (34.4)	36 (30.8)
Larynx	61 (25.5)	33 (27.0)	28 (23.9)
Oral cavity	53 (22.2)	23 (18.9)	30 (25.6)
Hypopharynx	10 (4.2)	5 (4.1)	5 (4.3)
Paranasal sinuses	6 (2.5)	3 (2.5)	3 (2.6)
Salivary glands	4 (1.7)	1 (0.8)	3 (2.6)
Lip and oral cavity	1 (0.4)	0 (0.0)	1 (0.9)
Nasopharynx	1 (0.4)	0 (0.0)	1 (0.9)
No primary site identified	4 (1.7)	2 (1.6)	2 (1.7)
More than one primary site	13 (5.4)	9 (7.4)	4 (3.4)
Other	8 (3.3)	4 (3.3)	4 (3.4)
Disease status at baseline; n (%)			
Eradicated, no recurrence	69 (29.7)	37 (31.4)	32 (28.1)
Eradicated, but recurred locally	107 (46.1)	49 (41.5)	58 (50.9)
Residual disease after prior therapy	48 (20.7)	27 (22.9)	21 (18.4)
Untreated	8 (3.5)	5 (4.2)	3 (2.6)
Unknown	7 (‐)	4 (‐)	3 (‐)
Received prior chemotherapy; n (%)	178 (74.5)	94 (77.0)	84 (71.8)
Received prior radiotherapy; n (%)	203 (84.9)	106 (86.9)	97 (82.9)
Had prior surgery; n (%)	146 (61.1)	70 (57.4)	86 (65.0)
Received prior biologic targeted therapy; n (%)	5 (2.1)	0 (0.0)	5 (4.3)

Abbreviations: ECOG, Eastern Cooperative Oncology Group; N, sample size; n, frequency; PS, performance status.

### 3.2 Internal consistency and test–retest reliability

See Table 2 for descriptive statistics of the PRO measures at each time point. Cronbach's alpha coefficients were satisfactory for FHNSI‐10 (range 0.68‐0.75) and FHNSI‐7 (range 0.60‐0.68) at all time points. In addition, among patients with stable ECOG PS from baseline to Week 4 (n = 123), test–retest reliability was acceptable for the FHNSI‐10 (ICC = 0.76) and FHNSI‐7 symptom indexes (ICC = 0.75).

**Table 2:** Summary statistics of the FHNSI‐10, FHNSI‐7, and individual symptom endpoints across time

PRO measure	Baseline				Week 2			
	N	M (SD)	Mdn	Mean (SD) change from baseline	N	M (SD)	Mdn	Mean (SD) change from baseline
FHNSI‐10	229	22.66 (6.35)	23	‐	179	22.17 (6.23)	23	−0.5 (4.9)
FHNSI‐7	229	15.78 (5.03)	16	‐	179	15.41 (4.77)	16	−0.4 (3.5)
Pain	229	4.48 (2.29)	5	‐	178	4.62 (2.21)	5	0.1 (1.9)
Fatigue	228	1.99 (1.13)	2	‐	179	1.74 (1.13)	2	−0.2 (1.2)
Swallowing	222	3.15 (2.44)	3	‐	178	3.19 (2.40)	3	0.0 (1.8)
Breathing	228	3.26 (1.02)	4	‐	178	3.18 (1.09)	4	−0.1 (1.0)
Communication	228	2.89 (1.32)	3	‐	177	2.72 (1.36)	3	−0.2 (1.4)
Symptom bother	212	0.93 (1.21)	0	‐	178	1.36 (1.15)	1	0.5 (1.5)

PRO measure	Week 4				Week 8			
	N	M (SD)	Mdn	Mean (SD) change from baseline	N	M (SD)	Mdn	Mean (SD) change from baseline
FHNSI‐10	166	23.23 (6.53)	23	0.2 (5.0)*	125	22.38 (6.74)	22	−0.9 (5.1)*
FHNSI‐7	166	16.04 (5.03)	16	−0.1 (3.9)	125	15.63 (5.06)	16	−0.6 (4.0)
Pain	162	4.96 (2.30)	5	0.3 (1.8)	124	4.94 (2.26)	5	0.3 (2.0)
Fatigue	166	1.87 (1.22)	2	−0.2 (1.3)	125	1.83 (1.13)	2	−0.2 (1.3)
Swallowing	163	3.17 (2.49)	3	0.0 (2.0)	123	2.90 (2.33)	2	−0.3 (2.1)
Breathing	166	3.22 (1.08)	4	−0.1 (1.0)	124	3.22 (1.06)	4	−0.1 (1.1)
Communication	166	2.81 (1.31)	3	−0.2 (1.5)	125	2.70 (1.36)	3	−0.2 (1.5)
Symptom bother	165	1.25 (1.14)	1	0.4 (1.4)	125	1.48 (1.24)	1	0.7 (1.6)

Notes

Possible ranges for each measure are as follows: FHNSI‐10, 0‐40; FHNSI‐7, 0‐28; Pain, 0‐8; Fatigue, 0‐4; Swallowing, 0‐8; Breathing, 0‐4; Communication, 0‐4, Symptom bother, 0‐4.

Abbreviations: FHNSI, Functional Assessment of Cancer Therapy‐Head and Neck Symptom Index; M, mean; Mdn, median; N, sample size; PRO, patient‐reported outcome; SD, standard deviation.

* Significant change from baseline with *p* < 0.05.

### 3.3 Known‐groups validity

We assessed known‐groups validity by examining the relationships between baseline PRO scores and patients’ baseline ECOG PS. Across almost all PRO measures, mean PRO scores for participants with an ECOG PS of 0 were significantly higher (better) than for participants with an ECOG PS of 1 or 2 (Table 3). As an exception, there was not a significant difference between the mean breathing scores of participants with ECOG PS of 0 and 1. For the individual symptom endpoint scores, we confirmed these conclusions using non‐parametric Kruskal‐Wallis tests.

**Table 3:** Summary statistics of the FHNSI‐10, FHNSI‐7, and individual symptom endpoint scores at baseline by baseline ECOG PS

Baseline PRO measure	Baseline ECOG PS						*p*‐value^†^	Significant pairwise comparisons^‡^
	0 (n = 27)		1 (n = 59)		2 (n = 143)			
	M (SD)	Mdn	M (SD)	Mdn	M (SD)	Mdn		
FHNSI‐10	28.11 (6.52)	27	22.19 (5.55)	23	21.82 (6.16)	22	<0.01	PS 0>PS 1 PS 0>PS 2
FHNSI‐7	20.19 (4.80)	20	15.29 (4.77)	15	15.15 (4.79)	15	<0.01	PS 0>PS 1 PS 0>PS 2
Pain	5.67 (2.37)	6	4.34 (2.24)	4	4.31 (2.24)	4	0.02	PS 0>PS 1 PS 0>PS 2
Fatigue	2.56 (1.01)	3	1.85 (1.08)	2	1.94 (1.15)	2	0.02	PS 0>PS 1 PS 0>PS 2
Swallowing	4.81 (2.37)	5	3.14 (2.39)	3	2.83 (2.35)	3	<0.01	PS 0>PS 1 PS 0>PS 2
Breathing	3.78 (0.51)	4	3.21 (1.10)	4	3.19 (1.04)	4	0.02	PS 0>PS 2

Notes

Possible ranges for each PRO measure are as follows: FHNSI‐10, 0‐40; FHNSI‐7, 0‐28; Pain, 0‐8; Fatigue, 0‐4; Swallowing, 0‐8; Breathing, 0‐4. The symptom endpoint for communication was excluded from this analysis, as we do not hypothesize that difficulty communicating is related to ECOG PS. ANOVAs were used to assess PRO score differences by ECOG PS, and non‐parametric Kruskal‐Wallis tests were used to confirm results of the ANOVA models for the individual symptom endpoints.

Abbreviations: ECOG, Eastern Cooperative Oncology Group; FHNSI, Functional Assessment of Cancer Therapy‐Head and Neck Symptom Index; M, mean; Mdn, median; PRO, patient‐reported outcome; PS, performance status; SD, standard deviation.

^†^ *p*‐value reflects the significance of the ANOVA model.

^‡^ Using Scheffe tests.

### 3.4 Predictive criterion validity

We assessed predictive criterion validity by examining the relationships between baseline PRO scores and anchor variables over time (i.e., change in ECOG PS from baseline to Week 4, OS, and TTP; Table 4).

**Table 4:** Univariate associations of the baseline FHNSI‐10, FHNSI‐7, and individual symptom endpoint scores with change in ECOG PS, overall survival, and time‐to‐progression

Baseline PRO measure	Change in ECOG PS			OS			TTP		
	N	Parameter estimate (SE)	*p*‐value	Number of events/N	HR (95% CI)	*p*‐value	Number of events/N	HR (95% CI)	*p*‐value
FHNSI‐10	177	<0.01 (0.01)	0.54	219/229	0.97 (0.95, 0.99)	<0.01	146/229	1.00 (0.97, 1.03)	0.97
FHNSI‐7	177	0.01 (0.01)	0.22	219/229	0.95 (0.93, 0.98)	<0.01	146/229	1.00 (0.97, 1.03)	0.88
Pain	177	<0.01 (0.02)	0.97	219/229	0.89 (0.84, 0.94)	<0.01	146/229	0.96 (0.89, 1.04)	0.32
Fatigue	176	−0.05 (0.04)	0.26	218/228	0.88 (0.77, 0.99)	0.04	145/228	0.93 (0.80, 1.08)	0.33
Swallowing	172	0.05 (0.02)	0.01	212/222	0.97 (0.91, 1.02)	0.23	141/222	1.04 (0.97, 1.12)	0.26
Breathing	176	−0.01 (0.05)	0.89	218/228	0.84 (0.74, 0.96)	0.01	145/228	0.98 (0.83, 1.16)	0.85

Notes

Associations of baseline PRO scores with change in ECOG PS were assessed using univariate general linear models, and associations of baseline PRO scores with OS and TTP were assessed using univariate Cox proportional hazards models. Univariate models were confirmed via multivariable models adjusted for age, sex, race, disease status, and prior treatments (not shown here). For models assessing the outcomes OS, and TTP, models were also adjusted for ECOG PS. The symptom endpoint for communication was excluded from these analyses, as we do not hypothesize that difficulty communicating is related to ECOG PS, OS, or TTP. Change in ECOG PS was defined as the value at Week 4 minus the value at baseline, so that positive change values indicate worsened functioning over time.

Abbreviations: CI, confidence interval; ECOG, Eastern Cooperative Oncology Group; FHNSI, Functional Assessment of Cancer Therapy‐Head and Neck Symptom Index; HR, hazard ratio; N, sample size; OS, overall survival; PRO, patient‐reported outcome; PS, performance status; SE, standard error; TTP, time‐to‐progression.

#### 3.4.1 Change in ECOG PS

Results from univariate general linear models showed that only higher (better) baseline scores for swallowing predicted increased (worsened) ECOG PS over time (*F*(1, 170) = 6.44, *p* = 0.01). All other associations did not reach statistical significance. These conclusions were confirmed via multivariable models.

#### 3.4.2 Overall survival

Results from univariate Cox PH models indicated that higher (better) baseline scores on the FHNSI‐10 index (HR = 0.97, 95% CI 0.95‐0.99, *p* < 0.01), the FHNSI‐7 index (HR = 0.95, 95% CI 0.93‐0.98, *p* = 0.01), and the pain (HR = 0.89, 95% CI 0.84‐0.94, *p* < 0.01), fatigue (HR=0.88, 95% CI 0.77‐0.99, *p* = 0.04), and breathing symptom endpoints (HR = 0.84, 95% CI 0.74‐0.96, *p* = 0.01) all significantly predicted lower risk of death. The association between swallowing and OS did not reach statistical significance. These relationships were confirmed via multivariable models, with the exception of fatigue; the relationship between fatigue and OS was no longer significant after adjusting for demographic and clinical variables.

#### 3.4.3 Time‐to‐progression

None of the baseline PRO scores significantly predicted TTP as evaluated by univariate or multivariable Cox PH models.

### 3.5 Responsiveness‐to‐Change

As seen in Table 2, there were few significant changes in PRO scores from baseline to Week 8. Exceptions were observed for the FHNSI‐10 (*F*(3, 231) = 3.34, *p* = 0.02) and fatigue scores (*F*(3, 231) = 3.12, *p* = 0.03). Specifically, FHNSI‐10 scores improved from Week 2 to Week 4 (*t*(231) = −2.09, *p* = 0.04) and worsened from Week 4 to Week 8 (*t*(231) = 2.59 *p* = 0.01). Fatigue scores worsened from baseline to Week 2 (*t*(231) = −2.88, *p* < 0.01) and also worsened from baseline to Week 4 (*t*(231) = −2.05, *p* = 0.04). All other changes in PRO scores did not reach statistical significance.

We used univariate ANOVA models to evaluate the relationships between changes in PRO scores and changes in ECOG PS from baseline to Week 4 (Table 5). Of the patients who completed the Week 4 PRO assessment, 69% (n = 103) had stable ECOG PS, 22% (n = 33) had improved ECOG PS, and 9% (n = 14) had worsened ECOG PS. Notably, of the 47 patients who had a change in ECOG PS, 87% of patients (n = 41) had a change of 1 point on the ECOG PS scale. Results of the ANOVA models showed that changes in PRO scores did not differentiate among patients with stable vs. improved vs. worsened ECOG PS. Kruskal‐Wallis tests further supported these conclusions.

**Table 5:** Change in PRO scores by change in ECOG PS from baseline to Week 4

PRO measure	Improved ECOG PS			No change in ECOG PS			Worsened ECOG PS			*p*‐value^†^
	N	M (SD)	Mdn	N	M (SD)	Mdn	N	M (SD)	Mdn	
Change in FHNSI−10	33	0.97 (5.31)	2.00	103	0.34 (4.66)	1.00	14	−2.14 (6.24)	0.00	0.14
Change in FHNSI−7	33	0.70 (4.01)	1.00	103	−0.08 (3.70)	0.00	14	−1.57 (5.21)	1.00	0.20
Change in Pain	32	0.69 (1.69)	1.00	100	0.16 (1.90)	0.00	14	0.43 (1.83)	0.00	0.36
Change in Fatigue	32	−0.09 (1.33)	0.00	103	−0.15 (1.22)	0.00	14	−0.29 (1.38)	0.00	0.89
Change in Swallowing	31	0.19 (2.18)	0.00	99	0.07 (1.98)	0.00	13	−1.00 (2.08)	−1.00	0.17
Change in Breathing	33	0.03 (0.95)	0.00	102	−0.05 (0.91)	0.00	14	−0.43 (1.87)	0.00	0.37

Notes

Change in the symptom endpoint score for communication was excluded from this analysis, as we do not hypothesize that difficulty communicating is related to ECOG PS. Change in ECOG PS was defined as the value at Week 4 minus the value at baseline, so that positive change values indicate worsened functioning over time. Change in PRO measure was defined as the value at Week 4 minus the value at baseline, so that positive change values indicate better functioning over time. Possible ranges for each PRO measure are as follows: FHNSI‐10, 0‐40; FHNSI‐7, 0‐28; Pain, 0‐8; Fatigue, 0‐4; Swallowing, 0‐8; Breathing, 0‐4.

Abbreviations: ECOG, Eastern Cooperative Oncology Group; FHNSI, Functional Assessment of Cancer Therapy‐Head and Neck Symptom Index; M, mean; Mdn, median; PRO, patient‐reported outcome; PS, performance status. SD, standard deviation.

^†^ *p*‐value reflects the significance of the ANOVA model.

### 3.6 Treatment side effect bother, PRO scores, and adverse events over time

Higher scores on item GP5 (“I am bothered by side effects of treatment”) have been associated with more clinician‐reported adverse events and with lower patient‐reported life enjoyment.^17^ Thus, we used univariate general linear models to explore whether this item was associated with the PRO scores, the incidence of adverse events, and with the maximum grade of adverse events over time (Table 6), and we confirmed the results using multivariable general linear models adjusting for age, sex, race, ECOG PS, disease status, and prior treatments.

**Table 6:** Univariate associations of item GP5 scores with the FHNSI‐10, FHNSI‐7, individual symptom endpoint scores, and adverse events over time.

PRO measure	Baseline			Week 4			Week 8		
	N	Parameter estimate (SE)	*p*‐value	N	Parameter estimate (SE)	*p*‐value	N	Parameter estimate (SE)	*p*‐value
FHNSI‐10	212	−0.03 (0.01)	0.049	165	−0.05 (0.01)	<0.01	125	−0.08 (0.01)	<0.01
FHNSI‐7	212	−0.02 (0.02)	0.29	165	−0.04 (0.02)	0.04	125	−0.10 (0.02)	<0.01
Pain	212	0.00 (0.04)	0.92	161	−0.09 (0.04)	0.03	124	−0.15 (0.05)	<0.01
Fatigue	211	−0.05 (0.07)	0.50	165	−0.21 (0.07)	<0.01	125	−0.41 (0.09)	<0.01
Swallowing	205	0.01 (0.03)	0.76	162	0.02 (0.04)	0.64	123	−0.08 (0.05)	0.08
Breathing	211	−0.20 (0.08)	0.01	165	−0.18 (0.08)	0.03	124	−0.43 (0.10)	<0.01
Communication	211	−0.11 (0.06)	0.08	165	−0.07 (0.07)	0.28	125	−0.13 (0.08)	0.12
Number of unique adverse events grade ≥1	212	−0.02 (0.03)	0.45	160	0.11 (0.04)	<0.01	119	0.08 (0.04)	0.06
Maximum grade of adverse events	212	0.00 (0.07)	0.96	160	0.14 (0.06)	0.03	119	0.16 (0.07)	0.02

Notes

Univariate general linear models were used to assess the relationships between the GP5 item, PRO scores, and adverse events at each time point. Univariate models were confirmed via multivariable models adjusted for age, sex, race, disease status, and prior treatments (not shown here).

Abbreviations: FHNSI, Functional Assessment of Cancer Therapy‐Head and Neck Symptom Index; GP5, single item reflecting treatment side effect bother; N, sample size; PRO, patient‐reported outcome; SE, standard error.

At baseline, higher GP5 (more treatment side effect bother) was significantly associated with lower (worse) FHNSI‐10 (*F*(1, 210) = 3.94, *p* = 0.049) and breathing scores (*F*(1, 209) = 6.02, *p* = 0.01). However, the relationship between GP5 score and the FHNSI‐10 was not sustained in a multivariable model. At Weeks 4 and 8, after patients initiated their assigned treatment, multiple associations emerged. Namely, higher Week 4 and Week 8 GP5 scores (more treatment side effect bother) were associated with lower (worse) FHNSI‐10, FHNSI‐7, pain, fatigue, and breathing scores. In addition, higher Week 4 GP5 was associated with more concurrent unique grade 1+ adverse events and with a higher maximum grade of adverse events. Higher Week 8 GP5 was also associated with a higher maximum grade of adverse events, but this relationship was not sustained in a multivariable model.

## 4 DISCUSSION

This study sought to validate the FHNSI‐10 and FHNSI‐7, two very brief patient‐reported symptom indexes, as well as individual symptom endpoints for use among patients with recurrent or metastatic SCCHN. We used data from an ECOG therapeutic trial (E1302) in which participants completed the FHNSI‐10^13^ plus one additional item related to treatment side effect bother (GP5) at multiple time points. Items from the FHNSI‐10 were used to compute the even briefer FHNSI‐7 and individual symptom endpoints for pain, fatigue, swallowing, breathing, and communication, each consisting of one or two items. The resulting symptom indexes and individual symptom endpoints included only the highest priority disease symptoms reported by patients with advanced head and neck cancer.^11^, ^12^

The FHNSI‐10 and FHNSI‐7 both performed adequately over time, with acceptable Cronbach's alpha internal consistency reliability coefficients over time and acceptable test–retest ICC reliabilities among patients with stable ECOG PS from baseline to Week 4. Of note, Cronbach's alpha is best suited as a measure of internal consistency reliability for scales that measure one latent construct as opposed to an index of various important elements (as is the case of the FHNSI‐10 and FHNSI‐7).^22^ Thus, our finding that Cronbach's alpha coefficients fell within the low range of acceptable internal consistency reliability is not considered a weakness of these indexes. In addition, ECOG PS was not assessed at Week 2. Thus, a shorter interval for test–retest reliability was not available, and stronger test–retest reliability might occur across intervals shorter than 4 weeks. The PRO measures showed known‐groups validity, as the FHNSI‐10, FHNSI‐7, and the pain, fatigue, and swallowing symptom endpoint scores successfully differentiated patients by provider‐rated ECOG PS 0 vs. 1 and 0 vs. 2 at baseline (pre‐treatment). In addition, the breathing symptom endpoint differentiated patients by ECOG PS 0 vs. 2, but was less sensitive to PS 0 vs. 1. The PRO measures were less successful in differentiating patients by change in ECOG PS over time; only better swallowing at baseline predicted worsened ECOG PS over time. This relationship is in the opposite direction that we would expect. However, it should be noted that change in ECOG PS was only calculated for a subset of the analyzable patient population, and our results should be confirmed in a larger sample of patients who experience a change in ECOG PS over time.

Similar to past work that has linked quality of life with survival, we found evidence of predictive criterion validity such that better scores on the FHNSI‐10 and FHNSI‐7 symptom indexes at baseline predicted better survival.^23^, ^24^, ^25^ Thus, these brief symptom indexes may have prognostic value among patients with recurrent or metastatic SCCHN. Moreover, less pain, fatigue, and breathing problems at baseline predicted better survival, though the relationship between fatigue and OS was not sustained after controlling for demographic and clinical variables. A recent review by Quinten and colleagues^26^ identified emotional functioning, nausea/vomiting, and dyspnea as specific aspects of quality of life that are particularly relevant for predicting survival in patients with head and neck cancer. Our findings provide additional support that breathing‐related symptoms provide prognostic information for patients with advanced head and neck cancer, particularly those with recurrent or metastatic SCCHN, and we extend prior work by identifying pain and possibly fatigue as other important markers of prognosis in this population. Interestingly, none of the PRO measures at baseline assessment predicted TTP, suggesting that factors other than patient health status may play a larger role in disease control.

We did not find evidence of responsiveness‐to‐change for the PRO measures by change in ECOG PS from baseline to Week 4. However, these null findings should be considered in the context of our data's limitations. As noted previously, longitudinal PRO data were only available for a subset of study participants. Moreover, the vast majority of patients with longitudinal data had stable ECOG PS and relatively few patients experienced changed ECOG PS. Nonetheless, for most PRO measures, mean changes were in the anticipated directions. Specifically, patients with improved ECOG PS tended to have positive PRO score changes, patients with worsened ECOG PS tended to have negative PRO score changes, and patients with stable ECOG PS tended to have minimal PRO score changes that fell between the two other groups. Future studies should consider assessing responsiveness‐to‐change for these PRO measures among a larger sample of patients in which a greater proportion of patients may experience changed ECOG PS.

Finally, we explored the associations between a single item that assesses how much patients are bothered by side effects of treatment and the PRO measures, the number of unique adverse events grade 1+, and the maximum grade of adverse events over time. At baseline, only more overall symptom burden on the FHNSI‐10 index and more breathing problems were related to more treatment side effect bother. However, after the initiation of treatment, more treatment side effect bother was associated with more overall symptom burden (FHNSI‐10 and FHNSI‐7) and more pain, fatigue, and breathing problems. Further, at Week 4, more treatment side effect bother was associated with more concurrent adverse events grade 1+ and with a higher maximum grade of adverse events. The association between more treatment side effect bother and higher maximum grade of adverse events persisted to Week 8. Our findings complement past work, which also found that worse treatment side effect bother was associated with higher maximum grade of clinician‐reported adverse events and less patient‐reported enjoyment in life across four clinical trials of various cancer populations.^17^ Our study provides additional support that a single item, “I am bothered by side effects of treatment,” could have value as a very brief, patient‐centered summary of treatment burden in clinical research and potentially clinical care.

Several limitations and considerations are noted. Although difficulty communicating is a high‐priority head and neck cancer‐related symptom,^11^, ^12^ our data did not include appropriate anchor variables by which to assess the validity or reliability of the communication symptom endpoint. Future studies should evaluate the psychometric properties of the communication symptom endpoint. Items assessing emotional functioning were not included in the symptom indexes or individual symptom endpoints evaluated here. This omission does not negate the importance of emotional functioning in this patient population. Rather, the brief symptom indexes and individual symptom endpoints evaluated here are meant to complement the assessments of other important aspects of the patient experience and provide options for very brief patient‐centered PRO assessments in cases in which patient burden must be minimized as much as possible. As noted, there was substantial attrition in this study's sample with regard to the completion of the PRO measures after the baseline assessment. Thus, the longitudinal findings should be interpreted with caution. Some findings did not conform to expectations, possibly due to conducting multiple comparisons. In addition, the sample predominantly identified as non‐Hispanic white, which limits the cross‐cultural generalizability of our findings. Generalizability to patients with diagnoses other than recurrent or metastatic SCCHN and patients with worse performance status (ECOG PS 3‐4) is also limited by parent study eligibility. Future work can evaluate the utility of these measures in expanded patient samples.

Nonetheless, these findings provide initial evidence for the validity for using the brief FHNSI‐10 and FHNSI‐7 symptom indexes and even briefer one to two item individual symptom endpoints (i.e., pain, fatigue, swallowing, and breathing) among patients with recurrent or metastatic SCCHN. These PRO measures may have value in clinical research and perhaps even clinical practice, and they can assist providers in conducting patient‐centered outcome assessments of patients with recurrent or metastatic SCCHN.

## CONFLICT OF INTEREST

DC and KW have ownership interests in FACIT.org. The other authors have no conflict of interest to disclose.

## AUTHOR CONTRIBUTIONS

AA and AF led the investigation of the larger clinical trial. LO, KW, and DC conceptualized the goals of this analysis. JWL performed data curation and formal analysis. LO and JWL performed data visualization. LO wrote the original manuscript draft with support from JWL, KW, and DC. All authors reviewed and edited the final manuscript.

## Data Availability

The data generated and/or analyzed for this paper are available at the NCTN/NCORP Data Archive (https://nctn‐data‐archive.nci.nih.gov/).
